# Black carbon aerosol number and mass concentration measurements by picosecond short-range elastic backscatter lidar

**DOI:** 10.1038/s41598-022-11954-7

**Published:** 2022-05-19

**Authors:** Romain Ceolato, Andrés E. Bedoya-Velásquez, Frédéric Fossard, Vincent Mouysset, Lucas Paulien, Sidonie Lefebvre, Claudio Mazzoleni, Christopher Sorensen, Matthew J. Berg, Jérôme Yon

**Affiliations:** 1 ONERA, The French Aerospace Lab, Toulouse University, 31055 Toulouse, France; 2 ONERA, The French Aerospace, Paris-Saclay University, CNRS, Laboratoire d’étude des microstructures, 92322 Châtillon, France; 3 ONERA, The French Aerospace Lab, 91761 Palaiseau, France; 4 Department of Physics, Kansas State University, 1228 N. 17th St., Manhattan, KS 66506-2601 USA; 5 Physics Department, Michigan Technological University, Houghton, MI USA; 6 CNRS, CORIA, INSA Rouen, UNIROUEN, Normandie University, 76000 Rouen, France

**Keywords:** Optical physics, Techniques and instrumentation, Characterization and analytical techniques, Climate sciences, Environmental sciences, Planetary science

## Abstract

Black carbon aerosol emissions are recognized as contributors to global warming and air pollution. There remains, however, a lack of techniques to remotely measure black carbon aerosol particles with high range and time resolution. This article presents a direct and contact-free remote technique to estimate the black carbon aerosol number and mass concentration at a few meters from the emission source. This is done using the Colibri instrument based on a novel technique, referred to here as Picosecond Short-Range Elastic Backscatter Lidar (PSR-EBL). To address the complexity of retrieving lidar products at short measurement ranges, we apply a forward inversion method featuring radiometric lidar calibration. Our method is based on an extension of a well-established light-scattering model, the Rayleigh–Debye–Gans for Fractal-Aggregates (RDG-FA) theory, which computes an analytical expression of lidar parameters. These parameters are the backscattering cross-sections and the lidar ratio for black carbon fractal aggregates. Using a small-scale Jet A-1 kerosene pool fire, we demonstrate the ability of the technique to quantify the aerosol number and mass concentration with centimetre range-resolution and millisecond time-resolution.

## Introduction

Black carbon (BC), as a component of particulate matter, is produced from the incomplete combustion of hydrocarbon fuel and biomass^1^. These particles consist of fractal aggregates of ultra-fine primary soot monomers, which are substantial climate-forcing agents due to their strong absorption of visible solar radiation in the atmosphere^2–4^. Black carbon emissions also influence the cloud formation processes which can impact regional circulation and rainfall patterns^5^. Moreover, these particles pose a threat to human health as they are considered a carcinogen and source of respiratory disease due to their nanometer size^6^; they also constitute a negative influence on urban air quality^7^. In other contexts, BC aerosols emitted by aircraft engines (also known as non-volatile particle matter) are potential ice nuclei and may induce cirrus clouds^8–11^. Yet, substantial uncertainties remain surrounding the net climate forcing of BC aerosols because of the large variety of substances encompassing freshly emitted as well as aged soot. Thus, the quantification of BC aerosol emissions, meaning aggregate-particle number $n_{\mathrm{o}}$ and mass concentration $m_{\mathrm{o}}$, is essential to advance our understanding of their role in both global warming and environmental health^12,13^.

A variety of techniques are available to characterize BC-aerosols such as filter-based absorption photometer^14^, photoacoustic measurements^15^, photothermal interferometry^16^, aethalometry^17^, or light-scattering principles^18^. For example, the Single Particle Soot Photometer (SP2) instrument employs laser-induced incandescence and light scattering^19^ as an *in-situ* technique to measure the size and volume fraction of BC particles^20^. The Scanning Mobility Particle Sizer (SMPS) can determine particle size and Soot Particle Aerosol Mass Spectrometry (SP-AMS) can provide on-line analysis BC-particle chemical compounds^21^. For all of these instruments and techniques, the measurements are local in nature, i.e., they do not provide at-a-distance range-dependent measurements and several of them are not widely used due to their cost and complex design^22^. Elastic backscatter lidar (EBL), however, is an active remote-sensing technique with the ability to characterize aerosols in a contact-free manner^23–26^. Such lidar operates by measuring laser light elastically scattered in the backward direction from an ensemble of particles. Until recently, EBL instruments mostly employ nanosecond pulsed laser sources to probe the atmosphere with several meter range resolution and several seconds to minutes time-resolution^27,28^. Generally, EBL instruments are rarely used for short-range applications due to an incomplete overlap between the outgoing laser beam and the receiver field-of-view. Environmental and air quality applications^29–35^ have recently raised a need for aerosol characterization close to the emission source, which is driving a decrease in the minimal measurement-range in EBL technology.

Here, we report on a novel remote-sensing EBL technique to quantify BC number and mass concentration. We demonstrate the feasibility of remote measurement of BC aerosols within the first tens of meters along the line of sight from the emission source with our instrument using a picosecond laser. These measurements feature a high degree of range and time-resolution. To our knowledge, there exist no published attempts to retrieve concentrations of ultrafine particulate matter, such as BC, via backscatter measurements with such high resolution close to the emission source. The following will present the PSR-EBL technique along with the Colibri instrument and a proof-of-principle measurement involving a Jet A1 kerosene pool-fire as a source for a BC aerosol. A dedicated lidar inversion method will be described that features an analytical model for lidar-relevant parameters (i.e. backscattering, and lidar ratio) based on the Rayleigh-Debye-Gans for Fractal-Aggregates (RDG-FA) theory. The results provided by our work should meet the growing need for BC particle measurements and could be assimilated into atmospheric transport models^36–38^, combustion-related issues for indoor^39^ or outdoor fires^40^, and health problems^41,42^.

## Results

### Principle of operation

**Figure 1:**
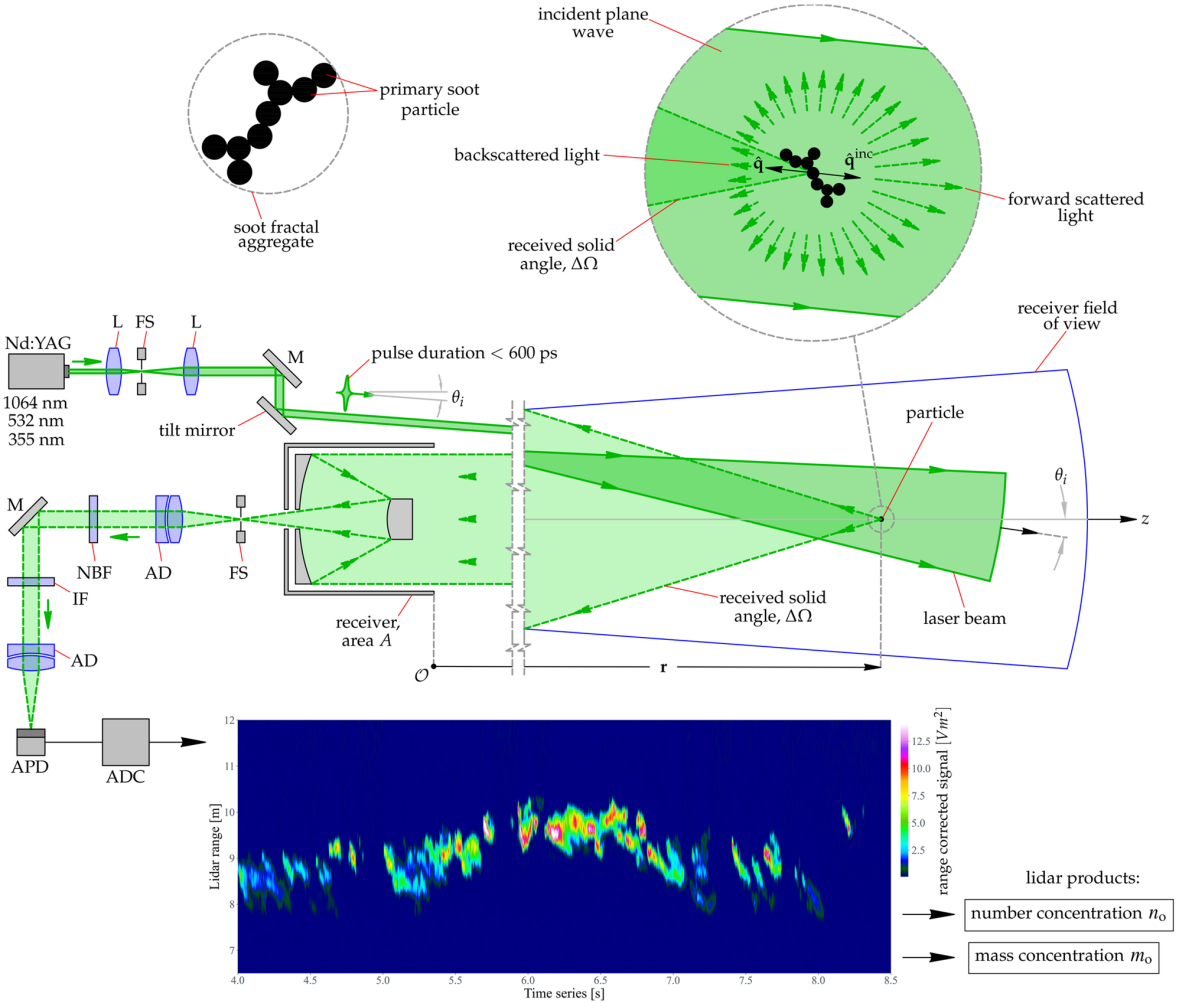
Principle of operation of the Picosecond Short-Range Elastic Backscatter Lidar (PSR-EBL) technique, intended to measure BC aerosol number and mass concentration, $n_{\mathrm{o}}$ and $m_{\mathrm{o}}$, respectively. A picosecond laser pulse is emitted from the lidar transmitter to illuminate a column of BC aerosols in the direction $\hat{\mathbf {q}}^{\mathrm{inc}}$. When a pulse arrives at a particle (shown inset) at a range *r*, it may be partly absorbed and will scatter in all directions $\hat{\mathbf {q}}$. The lidar return signal is directly related to the light backscattered by the particle to the receiver’s area *A*, which defines the received solid angle $\Delta \Omega$. An example measurement of the return signal is shown at the bottom for a small-scale kerosene pool-fire at a range of 9 m from the instrument. Further description of the Colibri lidar is given in the Methods section.

The Picosecond Short-Range Elastic Backscatter Lidar (PSR-EBL) is an active remote-sensing technique designed to measure the number and mass concentration profiles of ultrafine particulate matter in a range-resolved manner. In this study, the ultrafine matter is a BC aerosol. The principle of operation is illustrated in Fig. 1 and is described in further detail in the Methods section. Here, a series of picosecond pulses are emitted from the lidar transmitter, illuminating a column of aerosol particles in the $\hat{\mathbf {q}}^{\mathrm{inc}}$ direction. When a pulse arrives at a particle located at $\mathbf {r}$ as shown in the inset in Fig. 1, it may be partly absorbed and will scatter in all directions $\hat{\mathbf {q}}$. The return-signal consists of the portion of light backscattered to the lidar receiver’s area *A*, which defines the received solid angle $\Delta \Omega$ centered on the backscattering direction $-\hat{\mathbf {q}}^{\mathrm{inc}}$. The position of the particle relative to the lidar, $\mathbf {r}$, depends on the range *r* as $\mathbf {r}=r\hat{\mathbf {q}}^{\mathrm{inc}}$ as shown in Fig. 1. Then, the backscattered specific intensity $\mathbf {\widetilde{I}}^{\mathrm{bac}}(\mathbf {r},-\hat{\mathbf {q}}^{\mathrm{inc}},t)$ can be directly derived from the Radiative Transfer Equation (RTE)^26^ as,

1 $$\begin{aligned} \mathbf {\widetilde{I}}^{\mathrm{bac}} \left( \mathbf {r},-\hat{\mathbf {q}}^{\mathrm{inc}},t\right) = \frac{c\tau }{2}\; \mathbf {U}(\mathbf {r},t)\cdot \mathbf {\widetilde{I}}^{\mathrm{inc}} \left( \mathbf {r},\hat{\mathbf {q}}^{\mathrm{inc}}\right) , \end{aligned}$$

where *c* is the speed of light, $\tau$ is the laser pulse duration, $\mathbf {\widetilde{I}}^{\mathrm{inc}}(\mathbf {r},\hat{\mathbf {q}}^{\mathrm{inc}})$ is the incident specific intensity, and $\mathbf {U}(\mathbf {r},t)$ is the attenuated backscattering Stokes matrix. For the polarization insensitive measurements considered here, Eq. (1) can be simplified^26^ by a scalar version of $\mathbf {U}$ as

2 $$\begin{aligned} U(\mathbf {r},t)= n_{\mathrm{o}}(\mathbf {r},t) \left<Z_{11}\left( \hat{\mathbf {q}}^{\mathrm{inc}},-\hat{\mathbf {q}}^{\mathrm{inc}}\right) \right> \exp \left[ -2 \, n_{\mathrm{o}}(\mathbf {r},t)\int \limits _{0}^{r} \langle C^{\mathrm{ext}}\left( \mathbf {r}'\right) \rangle \,\mathrm{d}\mathbf {r}' \right] , \end{aligned}$$

where $n_{\mathrm{o}}(\mathbf {r},t)$ is the range and time dependent number concentration of particles, $\langle . \rangle$ is the ensemble-averaged operator, $Z_{11}(\hat{\mathbf {q}}^{\mathrm{inc}},-\hat{\mathbf {q}}^{\mathrm{inc}})$ is the first element of the Stokes phase matrix with units of area per solid angle, and $C^{\mathrm{ext}}$ is the extinction cross-section per particle with units of area.

The Colibri lidar is a forward-looking instrument based on the PSR-EBL technique. It employs a high repetition rate laser with picosecond pulses, which permits backscatter measurements with a millisecond time and centimeter range-resolution using the time-of-flight principle for distance determination. This is in contrast to conventional lidar systems intended for atmospheric studies. The instrument operated for several hours on February 20$^{\mathrm{th}}$, 2021 at an outdoor facility at ONERA in Occitanie, France. BC aerosols are generated from the combustion of a pool of aviation fuel (Jet A-1 kerosene), which is a sulfur-containing complex mixture of various hydrocarbons and alkanes. The small-scale pool fire generates plumes of soot at a range of 10 m from the Colibri system. The measurements were performed at 10 m laterally from the flames and at 1.3 m height. The efficiency of the pool-fire depends on several parameters including environmental conditions (wind, temperature, ambient pressure), and the BC $n_{\mathrm{o}}$ and $m_{\mathrm{o}}$ are continuously characterized in the experiment using an optical particle counter (Palas, Fidas 200). A first proof-of-principle of PSR-EBL technique is shown at the bottom of Fig. 1. Here, the range-corrected backscatter signals, which are directly related to the amount of BC aerosol, i.e., $n_{\mathrm{o}}$, are displayed for 4.5 seconds, at a distance of nine meters, and at a height of 1.20 meters above the pool-fire. A methodology will be described below to retrieve the range and time-dependent profiles of BC $n_{\mathrm{o}}$ and $m_{\mathrm{o}}$ from the return signals.

### Microphysics of the BC particles

**Figure 2:**
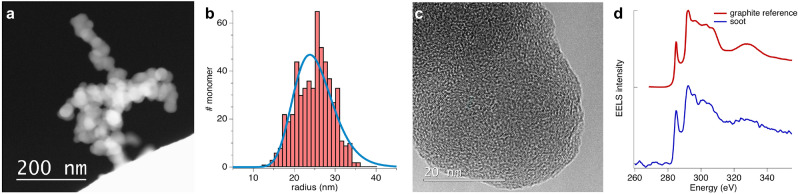
Microphysical properties of BC particles from a Jet-A1 pool-fire. In (**a**) is a STEM/HAADF image of a typical BC aggregate, while (**b**) shows the size distribution, in radius, of the monomers (red bars) and its lognormal fit (blue). In (**c**), a HRTEM image of a monomer is shown illustrating an onion-like structure and (**d**) presents the C–K edge EEL spectra of a monomer in blue and for graphite in red as a reference.

Using Scanning Transmission Electron Microscopy with a High Angle Annular Dark Field (STEM/HAADF) feature, BC aggregates collected on copper TEM grids at 1.2 meters above the Jet-A1 pool-fire are characterized as shown in Fig. 2. One can see that the BC particles consist of clusters of carbonaceous primary particles, or monomers, with a high fraction of graphite-like $\mathrm{sp}^{2}$-bonded carbon atoms. In the STEM/HAADF mode, electrons from a nanometric probe are elastically scattered by the particle nuclei and collected by an annular detector to form the image contrast seen. Incoherent scattering of this kind provides a simpler analysis of the image contrast by minimizing the dynamic effects that hamper conventional bright-field images. Consequently, the contrast values depend only on the number and type of atoms scattering the electrons. As such, the intensity collected by the image sensor can be directly linked to the thickness of the sample, assuming that the composition of the material is homogeneous^43^.

Figure 2a shows a STEM/HAADF image of a typical BC aggregate containing approximately 90-100 monomers. The monomers present a nearly spherical shape with a diameter smaller than 100 nm^3^ and form a fractal-like aggregate with a typical fractal dimension^44^ of $D_{\mathrm{f}}=1.8$ and up to hundreds of nanometers in size. The monomer size distribution, shown in Fig. 2b in terms of monomer radius, is obtained by measuring at least 550 monomers from 10 different aggregates (see examples in SI) and is fitted with a lognormal distribution. The mean radius is $R_{\mathrm{m}}=23.8 \pm 0.4$ nm and the mean number of monomers is $N_p=100$, which will be used later in the LIDAR inversion model. Using this distribution in combination with the aggregate size deduced from the contrast in the image and its projected area, the aggregate volume can be deduced. That information then estimates the average number of monomers and the surface area of the aggregate assuming that necking between the monomers is negligible. Further characterization of the monomers is shown by the high-resolution TEM (HRTEM) analysis in Fig. 2c where the onion-like structure with randomly-orientated fringes indicates a microstructure typical of carbon black with its turbostratic stacking^45–47^.

Electron Energy Loss Spectroscopy (EELS) is also performed at the carbon K-edge on several monomers. A typical EEL spectrum of BC is shown in blue in Fig. 2d. The shape of the edge presents several features which are well-known and related to $$\mathrm{sp}^{2}$$-hybridized carbon. Indeed, the first peak at 285 eV corresponds to the transition between the carbon 1s state and the first lowest unoccupied molecular orbital, which in this case is a $$\pi ^*$$ orbital. The second peak is related to the higher anti-bonding orbital $$\sigma ^*$$ of carbon. One tried to quantify the amount of $$\mathrm{sp}^{2}$$-hybridized carbon by EELS by studying the peak area ratio $$\pi ^*$$ to $$\pi ^*+\sigma ^*$$^48^. We used this criterion to estimate the amount of graphitic (aromatic cycles) versus amorphous carbon (C-H bonds) and it reveals a strong anisotropy of the graphitic structure combined with the spherical morphology of the monomers^49,50^. Additionally, the presence of an excitonic feature at 291.8 eV confirms the graphite-like nature of the material^51^.

### Number and mass concentration

The lidar return signals, which are related to Eq. (2), must be inverted to retrieve an estimate of the BC aerosol number and mass concentration, $n_{\mathrm{o}}$ and $m_{\mathrm{o}}$, respectively. While the details of this inversion are given in the Methods section, it involves lidar products obtained at three levels:

(i) The first-level products are the attenuated backscatter profiles $\mathrm{U}(r,t)$ of Eq. (2), which are the range corrected lidar signals resulting from the application of a radiometric calibration^52^. The lidar signals are pre-processed to increase the signal-to-noise ratio. Here, this pre-processing consists of a dark current correction (DC), a background correction (BG), and a low pass filtering method that preserves the range resolution of the original signal^53^.
(ii) The second-level products are backscatter profiles $\beta (r,t)$ obtained from a forward lidar-inversion method applied directly to the $\mathrm{U}(r,t)$ signals. The inversion uses a light-scattering model that accounts for the fractal morphology of BC aerosols and is an essential element in determining accurate backscatter profiles from PSR-EBL technique. Here, the lidar ratio is calculated using the Rayleigh-Debye-Gans for Fractal Aggregates (RDG-FA) theory and the microphysical parameters provided by the STEM/HAADF analysis.
(iii) Lastly, third-level products are the BC aerosols number and mass concentration range and time-dependent profiles $n_{\mathrm{o}}(r,t)$ and $m_{\mathrm{o}}(r,t)$. These are calculated using, respectively, the differential backscattering cross-section $\mathrm {d}C^{\mathrm{bac}}_{\mathrm{aer}}$ and mass-specific backscattering efficiency $\sigma ^{\mathrm{bac}}$ for BC fractal aggregates via RDG-FA theory.

**Figure 3:**
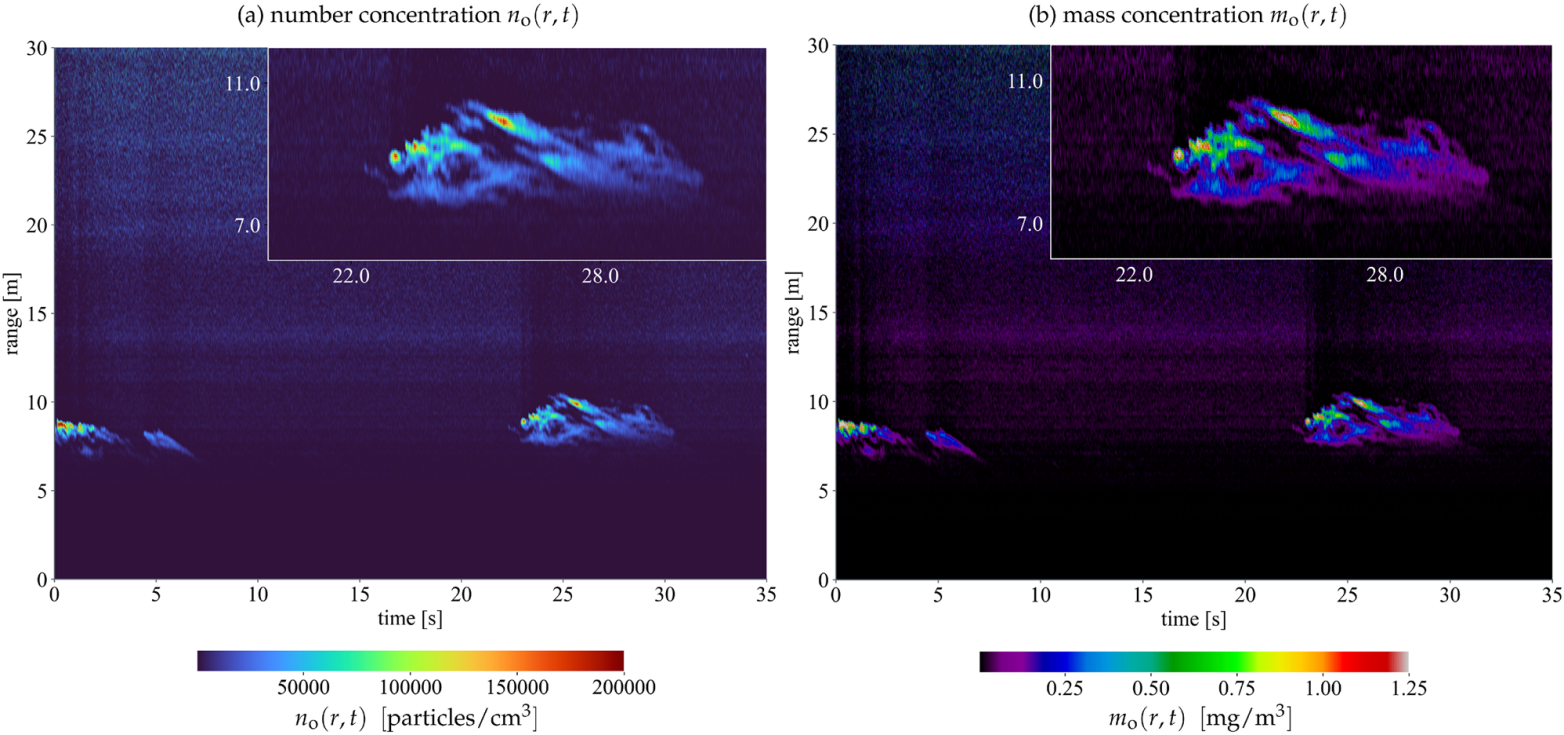
Range and time-resolved number $n_{\mathrm{o}}(r,t)$ and mass $m_{\mathrm{o}}(r,t)$ concentration profiles from the PSR-EBL technique of BC aerosols emitted by a small-scale Jet A-1 pool-fire. To highlight the resolution obtained, the inset images show a magnified view of the plume occurring between 20-30 s.

Examples of the third-level lidar products $n_{\mathrm{o}}(t,r)$ and $m_{\mathrm{o}}(t,r)$ are presented in Fig. 3. The measurements display two plumes of BC emitted from the pool-fire at approximately 9 m from the lidar instrument. These results demonstrate the ability of the PSR-EBL to perform contact-free measurements at a range-resolution of 5 cm and a time-resolution of 4 ms, which is revealed by the insets of the later-time plume in Fig. 3. The high spatio-temporal resolution of the PSR-EBL technique allows novel possibilities of measurements for such rapid and turbulent phenomena. Further investigations should be conducted using a stable kerosene flame in an aerosol chamber equipped with an extensive suite of state-of-the-art instruments to establish a more comprehensive assessment of the full capabilities of the PSR-EBL technique.

## Discussion

The findings of this study have important implications that overcome several limitations of conventional EBL techniques. One such limitation relates to lidar range and time resolution. Results from the Colibri instrument can be used to estimate the BC aerosol number and mass concentration with centimeter range-resolution and millisecond time-resolution, by virtue of the picosecond laser and fast return-signal sensor. A second limitation concerns the capability of measuring aerosol concentration in the short-range. Most EBL instruments are “blind” in the short-range due to an incomplete overlap between the emitted laser beam and receiver field of view. Here, however, we demonstrate that this limitation can be relived and concentration profiles obtained at ranges of 8-10 meters in the outdoor environment through a bi-static bi-axial lidar configuration. A third limitation concerns the retrieval methods needed to invert the return signals to retrieve the aerosol characteristics of interest. Common EBL inverse methods rely on assumptions such as an aerosol-free zone. While here the inverse method requires a prior radiometric calibration of the system, it does enable the accurate measurements in any environmental condition, without the need for a reference or a clean molecular zone in the atmosphere, i.e. molecular normalization^54^. And lastly, most inversion methods in remote-sensing rely on spherical or spheroidal proxies for the aerosol particle in calculating the aerosol lidar-relevant properties, i.e., the lidar ratio and backscattering cross-sections. Through the use of the RDG-FA theory, these quantities are pre-calculated and account for the fractal morphology of the BC particles, including such physically relevant parameters as monomer size and aggregate fractal dimension.

While BC particulate matter is typically characterized by local *in-situ* sensors, there is a growing need for short-range optical remote-sensing techniques^28,55^. Indeed, most sensors currently available do not provide range-dependent profiles as they rely on arrays of spatially distributed and time-integrating samplers to collect particles for analysis. A prominent advantage of active remote-sensing techniques, such as the PSR-EBL technique described here, is that they do not require spatial interpolation or aerosol-dispersion models as they directly provide the range and time-resolved measurements. However, a major challenge for these techniques is the need for an accurate aerosol-optic model for retrieving the number and mass-concentration of particles. Here, we have proposed to use a well-established light-scattering model in combustion science, i.e. RDG-FA, for assessing the lidar quantities for soot fractal aggregates. Other approaches may be used using an equivalent shape model^56–58^. The uncertainty of the optical and microphysical parameters is an additional difficulty to the methodology: for instance, the presence of organic coating may affect the fractal geometry of soot^59^ as well as its refractive index^60,61^. Nevertheless, considering all these uncertainties, the relative errors on the retrieved concentrations were estimated to be less than 28% for both number and mass concentration by an error propagation analysis (See Supplementary information for details), which is consistent with other similar studies^62,63^. Surely, further refinement of the aerosol-optic model will reduce the uncertainty of the methodology.

The results presented here demonstrate the potential of the PSR-EBL technique to estimate the range and time-resolved number and mass concentrations of BC emissions. While the high range and time-resolution and short-range capabilities provide a new approach in lidar for such particle measurements, further work is necessary to evaluate the full capabilities of the technique. Future studies could better assess the level of agreement between the PSR-EBL and other well-established techniques such as SMPS or SP-AMS. Future developments in PSR-EBL technique could provide improved insight for BC-aerosol emission studies, especially given that our work is subject to limitations, including the need for detailed microscopy of representative BC particles. As another improvement, a multi-wavelength picosecond laser could be used to infer more detail regarding the optical properties of BC-aerosol particles, including for example, the monomer particle-size, the presence of aggregate aging, coating, and even distinguishing black-carbon from brown-carbon aggregates. Finally, the PSR-EBL technique could really help in characterizing BC due to its high-spatial and time resolution. However, great care has to be taken in modeling the radiative lidar quantities as the accuracy of the retrieved products is tightly linked to the choice of the aerosol-optic model. Further investigations will have to be pursued for improving the aerosol-optic model for freshly emitted soot particles.

## Methods

### Short-range micro-lidar instrument

The Colibri instrument is a forward-looking picosecond short-range elastic backscatter lidar (PSR-EBL) with a bi-static, multi-axial architecture. The system was developed by ONERA, The French Aerospace Lab, for remote measurements of aerosols with high range and temporal resolution^26,52^. Compared to other lidar systems for atmospheric studies, Colibri is lightweight, compact, and suitable for a mobile platform. The transmitter unit is composed of a compact air-cooled Nd:YAG laser that emits 600 ps pulses with a pulse energy of 25 $\upmu$J, wavelength of $\lambda =532.8$ nm, repetition rate of 1 kHz, and a beam divergence of 0.5 mrad. As shown in Fig. 1, the linear-polarized beam is reflected and directed towards the aerosols of interest using a tilt mirror. A bi-static architecture is preferred over a mono-static configuration to prevent pulses from causing internal reflections that could saturate the sensor and thus reduce the minimum range of measurement. The bi-static angle, i.e. the angle $\theta _{i}$ subtended between the transmitter and receiver units, is an essential feature for short-range measurements as it enables full control of the overlap function. The receiver unit employs a Cassegrain telescope with a 90 mm effective diameter and 500 mm focal length along with field-stop (FS). Light collected by the telescope is focused on the sensor by a pair of achromatic doublet (AD) lenses, with a neutral-band filter (NBF) and interference filter (IF) to control light levels. For short-range measurements, the optical elements following the telescope are placed on a translation stage, allowing adjustment of the lidar focal plane. This feature is essential for short-range lidar measurements as it resolves focusing problems encountered for short ranges. For the measurements reported here, the focal plane position is set to maximize the collected signal magnitude at a range of approximately 10 m. The sensing unit is a high-bandwidth silicon avalanche photodiode (APD) and its analog signal is passed to a high bandwidth single-channel Digital Signal Processing (DSP) to digitize the signal.

### Inversion method

The elastic lidar equation can be derived from the Radiative Transfer Equation (RTE)^26^, which itself can be derived from first principles, i.e., from the Maxwell equations, as shown by Mishchenko^64^. The lidar equation usually assumes that the incident light is scattered only once, i.e., multiple-scattering events are ignored, and thus, can be analyzed as a link-budget for the backscattered power $\mathrm{P}(r)$ as a function of range *r* as:

3 $$\begin{aligned} \mathrm{P}(r)= \mathrm{K}_{\mathrm{o}}\,\mathrm{O}(r)\,\mathrm{U}(r)\,r^{-2} \end{aligned}$$

where $\mathrm{r}^{\mathrm{-2}}$ is the quadratic decrease due to solid angle of the lidar, $\mathrm{K}_{\mathrm{o}}$ is the radiometric lidar constant, $\mathrm{O}(r)$ is the range-dependent overlap function accounting for the partial overlap between the lidar field of view and the laser beam, and $\mathrm{U}(r)$ is the attenuated backscattering function defined as:

4 $$\begin{aligned} \mathrm{U}(r)= \beta (r)\exp \left( {-2\int _0^{r}\alpha (r')\,\mathrm {d}r'}\right) . \end{aligned}$$

with units of inverse distance time inverse solid angle. In Eq. (4), $\alpha =\alpha _{\mathrm{aer}}+\alpha _{\mathrm{mol}}$ and $\beta =\beta _{\mathrm{aer}}+\beta _{\mathrm{mol}}$ which represent the total extinction and backscattering coefficients as sums of the BC aerosol (bc) and the molecular components (mol), i.e., that due to the gas molecules in the atmosphere. The ill-posed nature of the lidar inversion problem requires the extinction-to-backscattering ratio, or lidar ratio (LR) to be assumed^65–67^. This ratio is defined for BC particles and molecular components, respectively, as $\mathrm{LR}_{\mathrm{aer}}=\alpha _{\mathrm{aer}}/\beta _{\mathrm{aer}}$ and $\mathrm{LR}_{\mathrm{mol}}=\alpha _{\mathrm{mol}}/\beta _{\mathrm{mol}}$.

We now describe a method to retrieve the BC backscattering coefficient directly from calibrated radiometric micro-lidar measurements. Equation (4) is converted to a form^68^ involving a single unknown, $\mathrm{LR}_{\mathrm{aer}}$. With $\mathrm{LR}_{\mathrm{mol}}$ regarded as known^69^, this form is obtained by splitting the exponential term into two parts so that only the total backscattering coefficient appears:

5 $$\begin{aligned} \mathrm{U}(r)&=\beta (r) \exp \left[ -2\int _0^{r}\mathrm{LR}_{\mathrm{aer}}\beta (r') \,\mathrm {d}r'\right] \exp \left[ {-2\int _0^{r}(\mathrm{LR}_{\mathrm{mol}}-\mathrm{LR}_{\mathrm{aer}}) \beta _{\mathrm{mol}}(r')\,\mathrm {d}r'}\right] . \end{aligned}$$

where $\beta _{\mathrm{mol}}$ is commonly predicted from Rayleigh theory using air density profiles. Thus, Eq. (5) can be further simplified via

6 $$\begin{aligned} \mathrm{V}(r)&=\beta (r) \exp \left[ -2\int _0^{r}\mathrm{LR}_{\mathrm{aer}} \beta (r') \,\mathrm {d}r'\right] \end{aligned}$$

with units of inverse distance time inverse solid angle. In Eq. (6), $\mathrm{V}(r)$ is a modified attenuated backscattering function, which is related to Eq. (5) as

7 $$\begin{aligned} \mathrm{V}(r)&=\mathrm{U}(r) \exp \left[ {2\int _0^{r}(\mathrm{LR}_{\mathrm{mol}}-\mathrm{LR}_{\mathrm{aer}})\beta _{\mathrm{mol}}(r') \,\mathrm {d}r'}\right] . \end{aligned}$$

Equation (6) now contains a single unknown, $\beta$, which yields an analytical solution ^52^ as:

8 $$\begin{aligned} \beta (r)=\beta _{\mathrm{aer}}(r)+\beta _{\mathrm{mol}}(r)= \frac{\mathrm{V}(r)}{1-2\,\mathrm{LR}_{\mathrm{aer}}\, \displaystyle \int _0^r \mathrm{V}(r')\,\mathrm{d}r'}. \end{aligned}$$

Equation (8), however, requires fine-scale evaluation of the exponential term in Eq. (7), which can become a source of growing numerical errors. The resolution adopted here is to simultaneously evaluate this term along with Eq. (6). Following Ceolato ^52^, two admittance quantities, *T*(*r*) and *W*(*r*), are introduced

9 $$\begin{aligned} \mathrm{T}(r)&= \frac{\mathrm{V}(r)}{\beta (r)}= \exp \left[ -2\int _0^{r}\mathrm{LR}_{\mathrm{aer}}\beta (r') \,\mathrm {d}r'\right] \quad \mathrm{and} \quad \mathrm{W}(r)= \frac{\mathrm{V}(r)}{\mathrm{U}(r)}= \exp \left[ -{2\int _0^{r}(\mathrm{LR}_{\mathrm{aer}}- \mathrm{LR}_{\mathrm{mol}})\beta _{\mathrm{mol}}(r') \,\mathrm {d}\mathrm{r}'}\right] . \end{aligned}$$

These can now be seen as solutions to the system of coupled first-order partial differential equations

10 $$\begin{aligned} \left\{ \begin{aligned} \partial _{r}\mathrm{W}(r)&= -2(\mathrm{LR}_{\mathrm{mol}}- \mathrm{LR}_{\mathrm{aer}})\beta _{\mathrm{mol}}(r)\mathrm{W}(r),\\ \partial _{r}\mathrm{T}(r)&= -2\mathrm{LR}_{\mathrm{aer}}\mathrm{U}(r)\mathrm{W}(r),\\ \mathrm{T}(0)&=\mathrm{W}(0)=1. \end{aligned}\right. \end{aligned}$$

The system in Eq. (10) is solved and $\beta _{\mathrm{aer}}$ is given by

11 $$\begin{aligned} \beta _{\mathrm{aer}}(r)= \frac{\mathrm{U}(r)\mathrm{W}(r)}{\mathrm{T}(r)}- \beta _{\mathrm{mol}}(r). \end{aligned}$$

Next, $\beta _{\mathrm{aer}}(r)$ is used to calculate $n_{\mathrm{o}}(r, R_g)$ and $m_{\mathrm{o}}(r, R_g)$, respectively, as:

12 $$\begin{aligned} \beta _{\mathrm{aer}}(r)= \int _{R_{\mathrm{min}}}^{R_{\mathrm{max}}} n_{\mathrm{o}}(r,R_g)\mathrm {d}C^{\mathrm{bac}}_{\mathrm{aer}}(r,R_g) \,\mathrm {d}R_g \end{aligned}$$

where $n_{\mathrm{o}}(r,R_g)$ is the particle number concentration per unit volume for an isotropic scattering medium formed by an ensemble of randomly oriented BC aggregates with radius of gyration $R_\mathrm{g}$. In Eq. (12), $R_{\mathrm{min}}$ and $R_{\mathrm{max}}$ are the minimum and maximum radii of gyration, and $\mathrm {d}C^{\mathrm{bac}}_{\mathrm{aer}}$ is the differential backscattering cross-section of a BC aggregate, which is defined as:

13 $$\begin{aligned} \mathrm {d}C^{\mathrm{bac}}_{\mathrm{aer}}(r,R_g)= \left. \frac{d\sigma ^{\mathrm{sca}}(r,R_g)}{d\Omega }\right| _{\theta =\pi } \end{aligned}$$

and has units of surface time inverse solid angle. For a given mass specific backscattering efficiency $\sigma ^{\mathrm{bac}}$, the mass concentration of BC particulate matter is^28,70^:

14 $$\begin{aligned} m_{\mathrm{o}}(r,R)= \frac{\beta _{\mathrm{aer}}(r)}{\sigma ^{\mathrm{bac}}}, \end{aligned}$$

with units of mass time inverse volume $\mathrm{mg}^{2}/m^3$. Note that $\sigma ^{\mathrm{bac}}$ is defined from the mass specific extinction coefficient $\sigma ^{\mathrm{ext}}$ and the lidar ratio for BC as:

15 $$\begin{aligned} \sigma ^{\mathrm{bac}}= \frac{\sigma ^{\mathrm{ext}}}{\mathrm{LR}_{\mathrm{aer}}} \end{aligned}$$

with units of surface time inverse solid angle and inverse mass ${[\mathrm{m}^{2}/\mathrm{(sr}\cdot \mathrm{mg)}]}$.

### Rayleigh-Debye-Gans for fractal aggregates theory

Several accurate electromagnetic scattering methods are available to simulate the radiative properties of BC aggregates in a numerically exact manner. Perhaps the most flexible is the Discrete Dipole Approximation^71^ (DDA). Yet, it remains difficult to implement such methods given the significant computational time required when they are used for lidar inversion. Thus, approximate models of the radiative properties remain justified. Here, the approximation pursued is the Rayleigh–Debye–Gans for Fractal Aggregates (RDG-FA) theory, which is shown to be accurate to model light-scattering of fractal aggregates ^72^, including lidar-relevant quantities^73^, and in controlled laboratory experiments^74^. Using the RDG-FA theory, the backscattering and extinction cross-sections can be simply and analytically derived, and then, used for the lidar inversion.

Because an aggregate’s monomers are small compared to $\lambda$, one can assume that the wave phase shift across a monomer is negligible; this is one aspect of the RDG-FA theory. Doing so is equivalent to assuming that the electromagnetic field within a monomer is uniform, which is valid for spherical monomers when $x_{\mathrm{m}}|m-1|\ll 1$ where $m=n+i\kappa$ is the complex refractive index and $x_{\mathrm{m}}=2\pi R_{\mathrm{m}}/ \lambda$ is the monomer size-parameter. In this case, the monomer will scatter in the so-called Rayleigh limit. The other assumption in RDG-FA theory is that across an aggregate, the monomers scatter the incident light independent of each other, i.e., monomer-monomer multiple scattering is neglected. We note that these assumptions have limitations and a summary is given at the end of this section. The RDG-FA theory then postulates that an aggregate’s absorption cross-section is the sum of the cross-sections for each monomer. While the condition $x_{\mathrm{m}}|m-1|\ll 1$ may be justified at the monomer level, it is not at the aggregate level due to the increased size resulting from the assemblage of many monomers. Thus, some care is needed to approximate the differential scattering cross-section since there can be a significant phase shift across the aggregate.

The RDG-FA theory can be derived from the Maxwell equations, which is done in the Appendix of Sorensen et al.^75^ In particular, the derivation highlights important aspects of the various approximations made. First, the assumption that the electromagnetic field is uniform within a given monomer is not strictly true. For a monomer radius of $R_{\mathrm{m}}=30$ nm, the largest we consider, the quantity $x_{\mathrm{m}}\left| m-1\right|$ used to motivate the RDG-FA treatment evaluates to $\sim 0.34$, and thus, does not necessarily meet the $x_{\mathrm{m}}\left| m-1\right| \ll 1$ criterion. Second, the assumption that monomer-to-monomer multiple scattering (within a given aggregate) is negligible is difficult to justify. Indeed, work by Sorensen^75^, Yon^76^, and Argentin^77^ use numerically exact simulations to show that multiple scattering is both present and wavelength dependent, as one would intuitively expect for monomers in physical contact in an aggregate. For example, in relation to the assumption of no monomer-monomer multiple scattering, Lu and Sorensen^78^ find evidence for such scattering in soot aggregates via depolarization measurements. Third, Sorensen et al.^75^ shows that the RDG-FA theory does not satisfy energy conservation when used to calculate an aggregate’s extinction cross-section via the optical theorem. Such observations present a paradox in that, despite its shortcomings, the RDG-FA does agree well with light-scattering measurements of BC fractal aggregates^44^, including backscattering^73^. The resolution of this paradox is explained by Berg^79^. In short, while the electromagnetic fields within the monomers are indeed not uniform, once the scattering properties of an aggregate are averaged over random orientations, the RDG-FA theory becomes a good approximation due to interference cancellations.

Provided that the lidar beam is vertically polarized and the received scattered light is also vertically polarized, the differential scattering cross-section $\mathrm {d}C^{\mathrm{sca,vv}}_{\mathrm{bc}}$ of a BC aggregate is proportional to the squared number of monomers $N_{\mathrm{m}}$, the scattering cross-section of an individual monomer $\mathrm {d}C^{\mathrm{sca,vv}}_{\mathrm{m}}$, and a function *f*, called structure factor, that accounts for the fractal structure of the aggregate. The structure factor depends on $R_{\mathrm{g}}$, the scattering angle $\theta$, and the aggregate’s fractal dimension $D_{\mathrm{f}}$, thus

16 $$\begin{aligned} \mathrm {d}C^{\mathrm{sca,vv}}= N_{\mathrm{m}}^2\,\mathrm {d}C^{\mathrm{sca,vv}}_{\mathrm{m}} f(R_{\mathrm{g}},\theta ,D_{\mathrm{f}}). \end{aligned}$$

We note that different expressions for *f* are reported in the literature^44,80^. Each formulation involves the scattering wave vector $q(\theta ,\lambda )=(4\pi /\lambda )\sin (\theta /2)$. Here, we use that formulated by Dobbins and Megaridis^81^ due to its simplicity and because it is known to be accurate at $\lambda =532$ nm even when internal monomer-monomer multiple-scattering within the aggregate is considered^76^:

17 $$\begin{aligned} f(R_{\mathrm{g}},\theta ,D_{\mathrm{f}})= \left\{ \begin{array}{l} \displaystyle \exp {\left[ \frac{-(qR_{\mathrm{g}})^{2}}{3}\right] } \ \,\,\mathrm{if}\,\,\ (qR_{\mathrm{g}})^{2}<\frac{3}{2}D_{\mathrm{f}} \\ \displaystyle \left[ \frac{3D_{\mathrm{f}}}{2e(qR_{\mathrm{g}})^{2}}\right] ^{\frac{D_{\mathrm{f}}}{2}}\ \,\,\mathrm{if}\,\,\ (qR_{\mathrm{g}})^{2} > \frac{3}{2}D_{\mathrm{f}} \end{array} \right. \end{aligned}$$

where it is understood that *q* is a function of $\theta$ and $\lambda$. For aerosols made of large clusters, only the power-law regime can be considered (second part of Eq. 17). The current expression is in good agreement with the amplitude of the power-law regime proposed by Heinson et al.^82^ but it must be noticed that amplitude may be affected by the aggregate polydispersity^44^. Based on these expressions, the simplest analytical expression for the backscattering cross-section can then be found as:

18 $$\begin{aligned} \mathrm {d}C^{\mathrm{bac}}_{\mathrm{aer}}= N_{\mathrm{m}}^2\frac{16\pi ^4 R_{\mathrm{m}}^6}{\lambda ^{4}}F(m) f^{\mathrm{bac}} C_\mathrm{p} \end{aligned}$$

where $F(m)=\left| (m^{2}-1)/(m^{2}+2)\right| ^2$, $f^{\mathrm{bac}}=f(R_{\mathrm{g}},\theta =\pi ,D_{\mathrm{f}})$ and $C_\mathrm{p}$ is a correction factor^83^ depending on the width of the aggregate-size distribution. When $C_{\mathrm{p}}=1$, the aggregates are monodisperse, which is the simplest case and the one we apply here. We note that Sorensen and Wang^83^ find that $C_{\mathrm{p}}=1.57$ for diffusion-limited cluster aggregates (DLCA) with $D_{\mathrm{f}}=1.75$. It is not clear what value for $C_{\mathrm{p}}$ applies to real-world BC aerosols in the atmosphere since their formation likely does not follow pure DLCA or reaction-limited cluster aggregation processes^84^. For this reason, we choose $C_{\mathrm{p}}=1$ and anticipate further refinement of the value form future backscattering measurements from real-world aggregates in the atmosphere.

An analytical expression of the lidar ratio can also be found as:

19 $$\begin{aligned} LR_{\mathrm{bc}}= \frac{\mathrm {d}C^{\mathrm{bac}}_{\mathrm{aer}}}{\mathrm {d}C^{\mathrm{ext}}}= \frac{\lambda ^3}{2\pi ^{2} N_{\mathrm{m}} R_{\mathrm{m}}^3f^{\mathrm{bac}}} \frac{E(m)}{F(m)}+\frac{8\pi }{3}\frac{g}{f^{\mathrm{bac}}} \end{aligned}$$

which has units of solid angle and where $E(m)=\mathrm{Im}\left\{ (m^{2}-1)/(m^{2}+2)\right\}$ and *g* is a correction factor also provided by Dobbins and Megaridis^81^ as:

20 $$\begin{aligned} g=\left[ 1+\frac{4}{3D_{\mathrm{f}}}\left( \frac{2\pi R_{\mathrm{g}}}{\lambda }\right) ^2\right] ^{-\frac{D_{\mathrm{f}}}{2}}. \end{aligned}$$

### Lidar-relevant quantities

The following section provides details about the optical and microphysical quantities used for calculating the lidar-relevant quantities used for retrieving the number and mass concentration, $n_{\mathrm{o}}$ and $m_{\mathrm{o}}$ from the RDG-FA theory.

The monomer radius $R_{\mathrm{m}}$ of BC monomers is typically $\sim 5-30$ nm, the number of monomers per aggregates $N_\mathrm{m}$ ranges between a few tens to a few hundreds, and the fractal dimension, used in the evaluation of $f^{\mathrm{bac}}$ and *g*, is typically $D_{\mathrm{f}}=1.8$. The radius of gyration $R_{\mathrm{g}}$ is a measure of overall aggregate size and can be estimated from the fractal scaling law^44^. All of these parameters can be determined from electron microscopy analysis^85^. While the refractive index of kerosene soot remains an open discussion in the literature, the composition of the fuel and the presence of volatile organic compounds in the combustion should be accounted in the choice of refractive index. In particular, organic coatings may be present on the soot particles and impact the refractive index of the emitted soot particles. Here, we used the refractive index model proposed by Kelesidis^61^, which depends on the soot composition based on its organic (OC) and elemental carbon (EC) content. For instance, the refractive index was found to be $m=1.66+i0.76$ for $OC/EC=0$ (uncoated soot) and $m=1.6219+i0.61$ for $OC/EC=0.1$ (thinly coated soot). These values of refractive index are close to the one reported by Chang and Charalampopoulos^86^, which has been used in several works for modeling and predicting radiative properties of black carbon from kerosene flame and pool fires^87–89^. Thus, the differential backscattering cross-section and lidar ratio used for the retrieval of $n_{\mathrm{o}}$ and $m_{\mathrm{o}}$ are $\mathrm{d}C_{\mathrm{bac}}=6.4\pm 1.5\,\times 10^{-4}\,\upmu \mathrm{m}^{2}\mathrm{sr}^{-1}$ and $\mathrm{LR}=131.1\pm 18.6\,\mathrm{sr}$, respectively. The mean values with corresponding standard deviation were estimated using the RDG-FA model and Monte-Carlo uncertainty analysis method, described in the Supplementary information. These values are consistent with the values reported in the literature for freshly emitted soot particles^28,90,91^. Regarding the mass specific extinction coefficient, Mulholland^92^ reports an averaged value of $\sigma ^{\mathrm{ext}}=8.7 \pm 1.1$ $\mathrm{m}^{2}/\mathrm{g}$ which is consistent with the results reported by Liu^4^ for mature BC aerosols. For the molecular component, $\beta _{\mathrm{mol}}$=1.51 $\times 10^{-6} ~ \mathrm{m}^{-1}\mathrm{sr}^{-1}$ was calculated using the following atmospheric conditions : 287.15 K for temperature, 80$\%$ relative humidity, 991.2 hPa for pressure, and $\mathrm{CO}_{\mathrm{2}}$ concentration up to 385 ppmv.

## Supplementary Information

Supplementary Information.

## Data Availability

Measured data supporting the findings of this study and the experimental results shown in Figs. 1, 2 and 3 are available from the authors upon request.
