# Supplementary material for: Black carbon aerosol number and mass concentration measurements by picosecond short-range elastic backscatter lidar
Source: Sci Rep. 2022 May 19;12:8443. doi: 10.1038/s41598-022-11954-7 (PMC9120489; doi:10.1038/s41598-022-11954-7)
Supplement: Supplementary file 1 — Supplementary Information. [file 41598_2022_11954_MOESM1_ESM.pdf]

Supplementary Information 1 - Kerosene Jet-A1 black carbon

This section reports several supplementary information regarding the Transmission Electron Microscopy measurements of kerosene Jet-A1 soot fractal aggregates collected during the experiment.

Black carbon morphology from Transmission Electron Microscopy

In Figure 1, several STEM/HAADF images are displayed from a Zeiss Libra 200 MC Transmission Electron Microscope operating at 200 kV either in conventional TEM (CTEM) or Scanning TEM (STEM). This microscope is equipped with an electrostatic omega monochromator and an in-column filter. The microscope delivers a point resolution of 0.23 *nm* and an information limit below 0.14 *nm*. Moreover, a slit inserted in the monochromator allows to reduce the chromatic aberration and to reach an energy dispersion of 0.4 eV. It is also equipped with a HAADF detector for Z-contrast imaging. The use of a HAADF detector is very convenient to form images with contrasts related to the chemistry and the thickness of the materials.

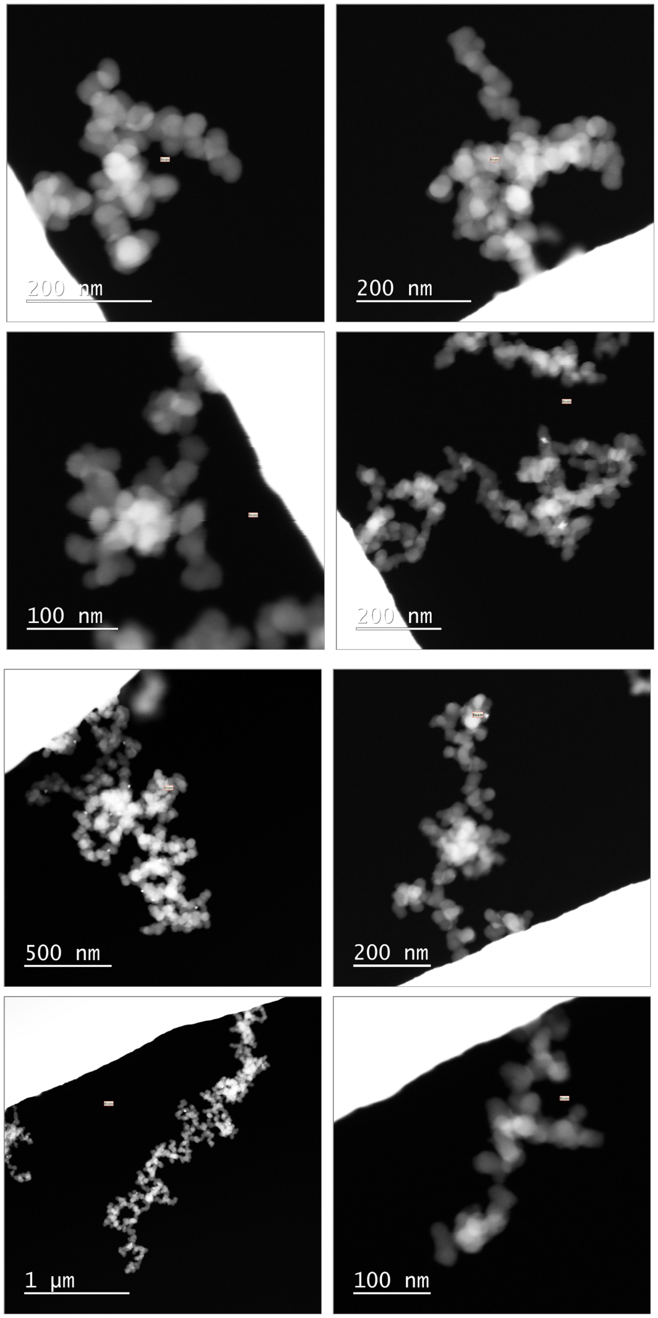

Figure 1. HAADF images of black carbon aerosols collected during the Jet-A1 small-scale pool fire experiment.

Figure 2 shows a profile analysis of the contrasts which confirm that a spherical profile is a good approximation of the monomer morphology and that, by carefully choosing the convenient dynamics of the detector, one can deduce the total amount of matter at the origin of the contrast assuming that the composition of the monomer and the overall soot aggregate is homogeneous.

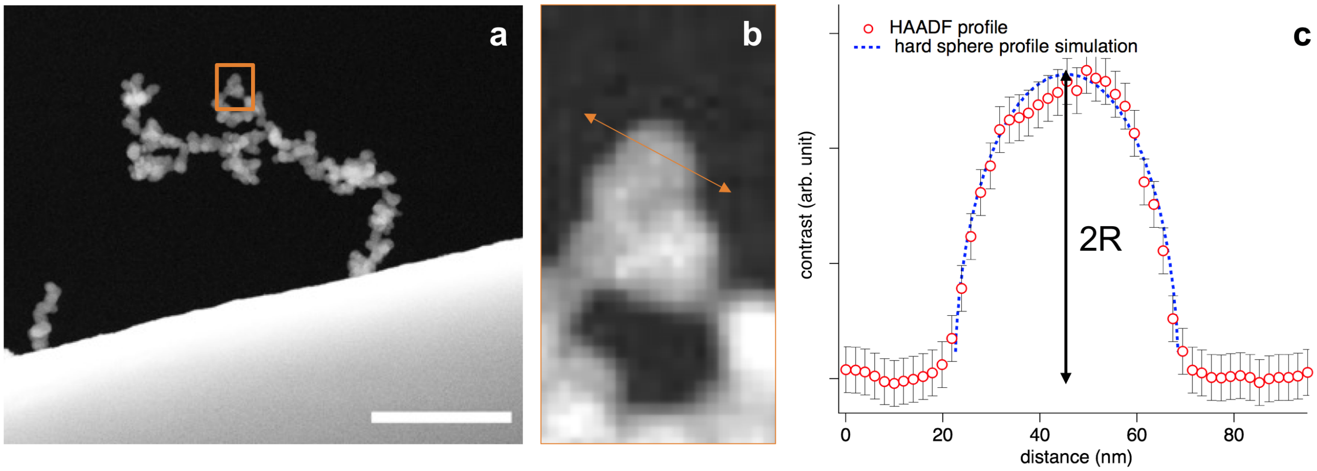

Figure 2. (a) image of a soot aggregate in HAADF mode - (b) zoom on a monomer for contrast profile extraction - (c) comparison between the contrast profile along the monomer and a projected hard sphere model

Black carbon chemistry from Electron Energy Loss Spectroscopy

Electron Energy Loss Spectroscopy (EELS) is a powerful tool to study chemistry and the chemical bonds between different atoms. In that case, the technique is even more relevant to study soot since it is more efficient for light atoms. Thanks to the abundant literature on carbon K-edge spectroscopy, one can distinguish amorphous carbon (green curve) from graphite (red curve), graphene (blue curve), or diamond (black curve) as illustrated in Figure 3. The main difference relies on the presence of π^* peak at 285 eV or σ^* peak at 294 eV which are related to different bounding configurations of carbon atoms.

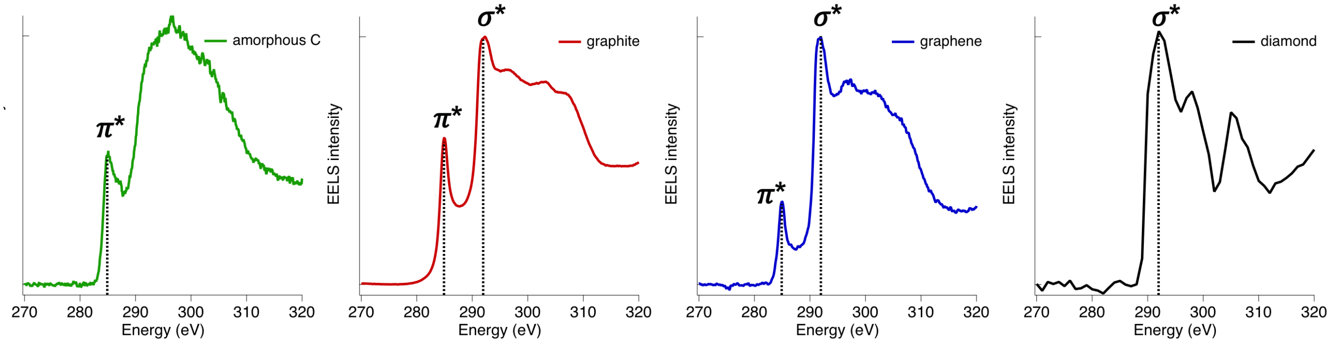

Figure 3. From left to right : EELS spectra of amorphous carbon, graphite, graphene and diamond

Supplementary Information 2 - Measurements processing

This section reports several supplementary information regarding the PSR-EBL pre-processing and processing procedures for retrieving the black carbon products, including number and mass concentration.

Retrieval scheme

The lidar retrieval scheme uses as input the attenuated backscatter $U(r)$, which is the first level product from the signal pre-processing detailed below. The lidar retrieval procedure requires a calculation of the LR, here using the RDG-FA theory. This LR along with $U(r)$ profiles are the inputs of the forward inverse method, described in the principal manuscript, to retrieve the aerosol backscatter $\beta_{\text{aer}}(r)$ profiles. As it is shown in Figure 4, the RDG-FA model and forward inverse method are used to estimate the number and mass concentration, n_0 and m_0 respectively.

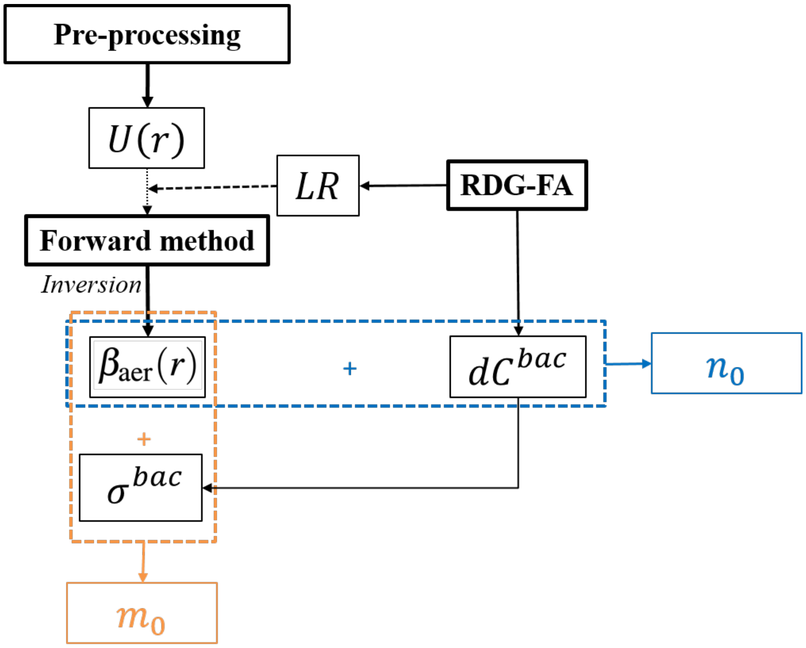

Figure 4. Block diagram of the lidar retrieval scheme.

Lidar pre-processing

Lidar pre-processing is a multi-step procedure aimed at improving the signal-to-noise ratio (SNR) of measurements. It is required to clean the signal from different kinds of noises (e.g. electronic, background). In this work, the procedure involved the following three steps : (i) electronic noise suppression, (ii) environmental or background noise suppression, and (iii) signal filtering. The first two steps are routine measurements carried out as a part of a pre-defined measurement protocol. Electronic noise measurements were done by keeping the laser on, but covering the other lidar sub-systems, assuring that the noise is only originated from the laser and/or the detectors. This type of measurement is usually referred to as dark current noise (DC).

Figure 5 presents the averaged 10min-DC profile in the first 10m showing that mean values are up to 4.6 mVm^2. Relatively high DC noise levels are seen in the first two meters, nevertheless, the order of magnitude of the DC signal compared with the mean raw profile (see on Figure 6 upper panel) shows the good performance of the APD detector. In the bottom of Figure 5, the temporal DC noise distribution allows distinguishing between any internal or external noise sources that can affect the measurements. Here, we selected the last 30 m (500 bins) to take the temporal mean values, checking that DC noise is well-behaved following a Gaussian-like distribution. The background (BG) noise was measured by selecting the same bin range of the signal (last 30 m), during a typical lidar measurement (without covering the telescope), determining the effect of the environmental light contamination. Figure 6 displays the lidar signal pre-processing procedure, starting from the raw signal (Panel 1) in the top of the figure and ending with the attenuated backscatter $U(r)$ (Panel 4). The rcs unprocessed (Panel 2) is the range corrected raw unprocessed signal, i.e. multiplied by the square of the range. The pre-processing steps described above are applied to produce the processsed rcs signal (Panel 3). Lastly, the signal is filtered by using a low-pass filter to reduce the remaining noises due to low frequencies. This filter can be seen in cyan solid line (Panel 4) which is not influencing the signal shape, on the contrary, it reduces the noise to prepare $U(r)$ signal to be inverted.

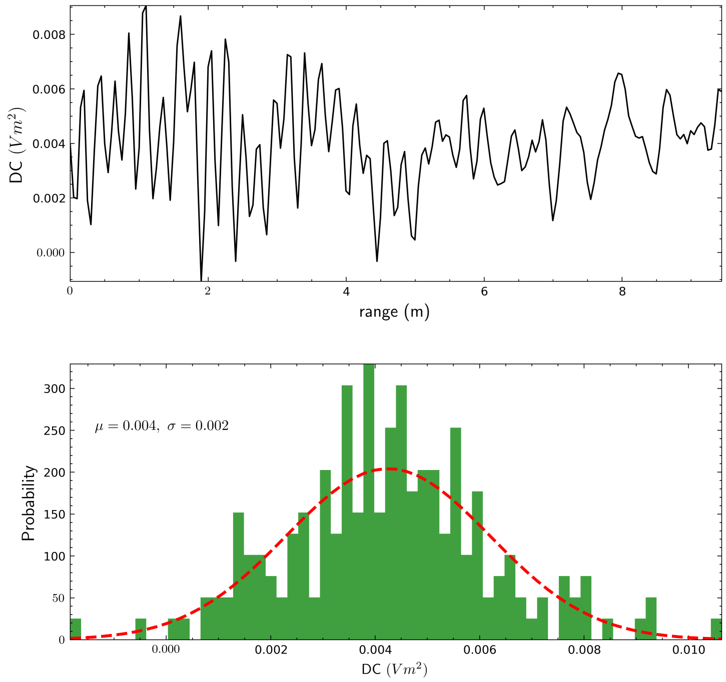

Figure 5. Dark current noise measurements. In the upper part of the figure a 10-min averaged DC profile is presented, while the temporal distribution of this noise is shown in the bottom

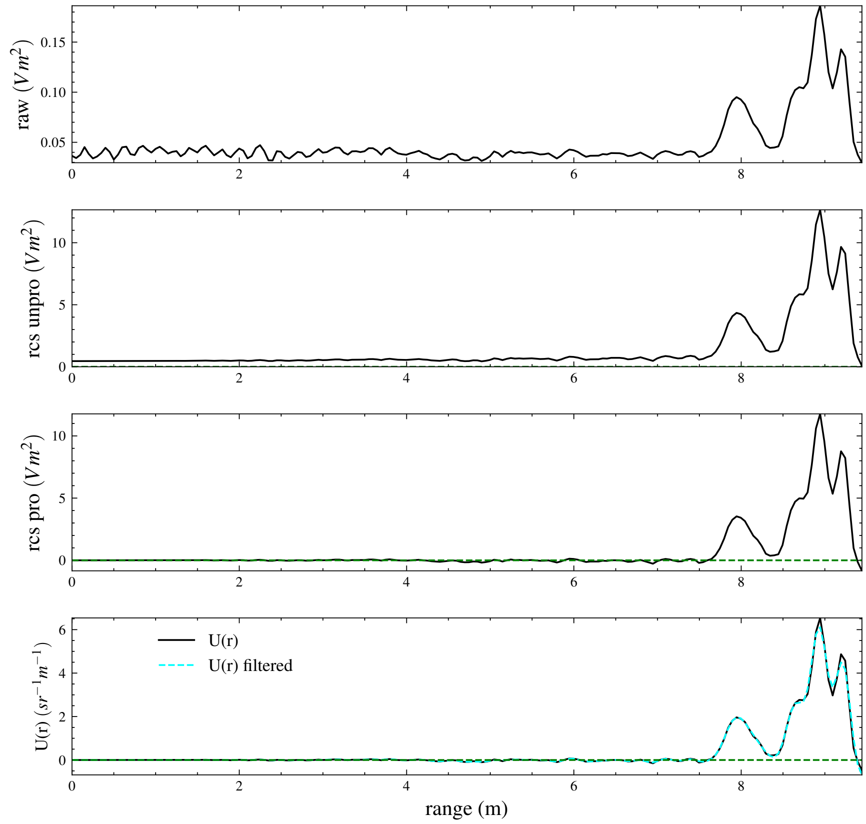

Figure 6. Lidar pre-processing. The first two panels refer to the raw and rcs signals, while the last two panels show the noise correction and signal filtering respectively. The $U(r)$ signal is the one used for further lidar inversion.

Supplementary information 3 - Error assessment and analysis

This section reports several supplementary information regarding the uncertainty analysis using a propagation model for the lidar products derived from the RDG-FA theory and the lidar inverse method.

Uncertainty analysis for lidar quantities from the RDG-FA theory

An uncertainty analysis based on a Monte Carlo (MC) approach is proposed in the following to quantify the error propagation of physical models, here the RDG-FA theory. This is done by assuming that the optical and microphysical variables are normally distributed with an assigned standard deviation and sampled over more than 1000 samples. Here, the input variables are the optical and microphysical parameters of the RDG-FA model with its respective set values for the simulations:

1. N_{m} : number of monomers (100 ± 10)
2. R_{m} : monomers radius $(23.8 \pm 0.3$ nm)
3. D_{f} : fractal dimension for aggregates (1.8 ± 0.5)
4. m : complex refractive index from Kelesidis et al.[1] accounting for the soot composition, *i.e.* ratio between organic carbon (OC) and elemental carbon (EC) ranging from 0 (non-coated soot with $1.66 + i0.76$) to 0.10 (thinly coated soot with $1.6219 + i0.6066$).

and the output results are the retrieved lidar quantities are :

1. $\mathrm{d}C_{\mathrm{bac}}$: differential backscattering cross-section
2. LR : lidar ratio, or extinction-to-backscattering ratio

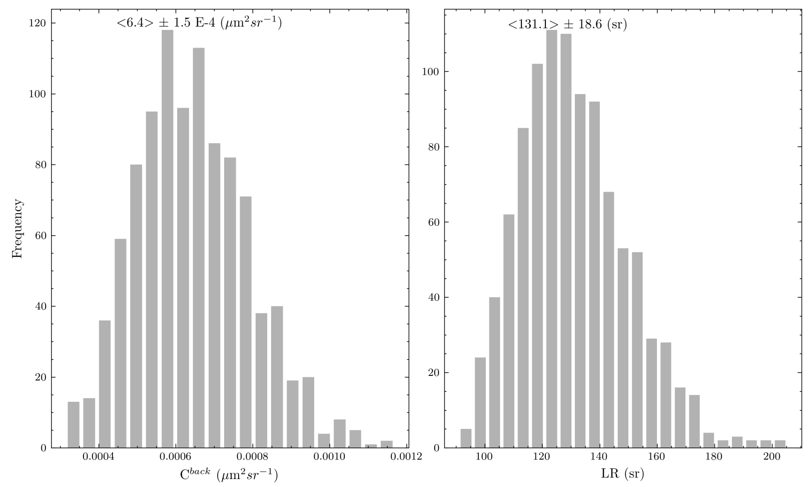

Figure 7. Monte Carlo simulation for evaluate the sensitivity of the RDG-FA model by changing the input parameters (m, and microphysical parameters of the soot monomer) for 1000 number of samples

Figure 7 presents the frequency distributions for the output quantities $\mathrm{d}C_{\mathrm{bac}}$ and LR obtained from the MC simulations. These results account for the uncertainties related to N_{m}, R_{g}, D_{f}, and m (from non-coated to thinly coated). It reports that the mean value of $\mathrm{d}C_{\mathrm{bac}}$ is to $6.4 \pm 1.5, \times 10^{-4} \mu\mathrm{m}^2\mathrm{sr}^{-1}$ and the mean value for LR is 131.1 ± 18.6 sr, which are consistent with the values reported in the literature for soot fractal aggregates[2] or even for fresh smoke[3,4]. These values of lidar parameters are used in the main manuscript for the retrieval of $n_{\mathrm{o}}(r,t)$ and mass $m_{\mathrm{o}}(r,t)$ concentration profiles from the Colibri PSR-EBL.

Validity of single-scattering lidar equation

A major source of uncertainties regards the correct use of the lidar equation, which remains valid only for single-scattering. An estimation of the contribution of multiple-scattering to the lidar signal has been performed using a stochastic lidar model[5,6]. This method consists in simulating the emission and transport of laser pulses in a scattering medium. The multiply scattered return lidar signal is computed by analysing the backscattered light reaching the receiver of the lidar instrument. The output of the lidar signal simulation code takes the form of a Stokes vector $\boldsymbol{S}$ for each ith scattering order, such as :

$$\boldsymbol{S}_i = \frac{A}{r^2}\frac{c\tau}{2}\beta_{aer}(r)\exp\left[-2\int_O^r \alpha_{\mathrm{aer}}(r')dr'\right]\boldsymbol{S}_i^{inc} \tag{1}$$

where A is the lidar telescope effective area, and $\boldsymbol{S}^{inc}$ is the Stokes vector of the incident electromagnetic wave on the telescope expressed using the well-known formula $\boldsymbol{S}_i = \begin{bmatrix} I_i & Q_i & U_i & V_i \end{bmatrix}^T$ [7]. The computation of the scattering events requires the knowledge of the Stokes phase matrix $\boldsymbol{Z}(\theta)$ of randomly-oriented soot fractal aggregates, which is computed as :

$$\boldsymbol{Z}(\theta) = N_\mathrm{m}^2 \frac{8\pi^4 R_\mathrm{m}^6}{\lambda^4} F(m) f(\theta) \begin{bmatrix} 1+\cos\theta^2 & \cos\theta^2 - 1 & 0 & 0 \\ \cos\theta^2 - 1 & 1+\cos\theta^2 & 0 & 0 \\ 0 & 0 & 2\cos(\theta) & 0 \\ 0 & 0 & 0 & 2\cos(\theta) \end{bmatrix} \tag{2}$$

The backscattering and extinction coefficients profiles, $\beta_\mathrm{aer}(r)$ and $\alpha_\mathrm{aer}(r)$ respectively, are averaged over the whole pool fire plume, and used as input parameters for the stochastic lidar model to quantify the impact of multiple-scattering. The multiple scattering fraction expresses the fraction of the simulated signal which is attributed to multiple scattering effects. It is expressed as :

$$MSF = 1 - \frac{I_1}{\sum_{i=0}^{\infty} I_i} \tag{3}$$

where i refers to the multiple scattering order. The lidar instrument characteristics are also modeled using the parameter values presented in the Method section. Using this procedure, the multiple scattering fraction within the plume is found to be lower than 0.1% for all the simulated profiles. This quantification of the multiple-scattering contribution to the lidar signal supports the use of the single-scattering approximation during the signal inversion process.

Error propagation for lidar inversion products

The error propagation for a single lidar profile was performed by assuming the noise of the variables as uncorrelated, and considering a certain degree of dependency between variables since calibration parameters namely $\mathrm{K_o^*}$ and $\mathrm{O(r)}$ were obtained using the same instrumental setup of the measurements. The ambient fluctuations are considered as an additional source of noise since its affects the measurements, producing statistical and ambient changes overtime; however, to minimize that during the calibration procedure, we used lidar measurements over a rather long period of time (more than 100 000 measurements) of the same Lambertian surface to improve the reliability of the inverse method.

Below are presented the equations involved in the error propagation procedure for each level of the lidar products:

$$\delta\mathrm{U(r)} = \left[\frac{\delta\mathrm{RCS}}{\langle\mathrm{RCS}\rangle} + \frac{\delta\mathrm{K_o^*}}{\langle\mathrm{K_o^*}\rangle} + \frac{\delta\mathrm{O(r)}}{\langle\mathrm{O(r)}\rangle} \right] \mathrm{U(r)} \tag{4}$$

where $\delta\mathrm{K_o^*}$ and $\delta\mathrm{O(r)}$ are the uncertainties associated to the calibration quantities $\mathrm{K_o^*}$ and $\mathrm{O(r)}$, and RCS that considers the error due to DC and BG. To propagate the error in β_aer, we considered in one hand that β_aer is at least two orders of magnitude larger than β_mol in our case, and on the other hand that β_mol remained quite stable during the measurements since atmospheric variables such as pressure, temperature and relative humidity did not change drastically. According to that, it is possible to neglect the influence of the β_mol on the error propagation. Thus, the error propagation of β_aer can be calculated by applying the partial derivative of the β_aer with respect to the $\mathrm{LR_{aer}}$ and U(r) as follows

$$\delta\beta_\mathrm{aer}(\mathrm{r}) = \left[(1 + 2\mathrm{LR_{aer}}\, \beta_\mathrm{aer}(\mathrm{r})\mathrm{U(r)}/\mathrm{U'(r)}) \frac{\delta\mathrm{U(r)}}{\mathrm{U(r)}} \right] \beta_\mathrm{aer}(\mathrm{r}) \tag{5}$$

Lastly, the error propagation for the number and mass concentration includes β_aer, dC^bac, and σ^bac errors

$$\delta n_\mathrm{o}(\mathrm{r,R}) = \left[\frac{\delta\beta_\mathrm{aer}(\mathrm{r})}{\langle\beta_\mathrm{aer}(\mathrm{r})\rangle} + \frac{\delta dC^{bac}}{\langle dC^{bac}\rangle} \right] n_\mathrm{o}(\mathrm{r,R}) \tag{6}$$

$$\delta m_\mathrm{o}(\mathrm{r,R}) = \left[\frac{\delta\beta_\mathrm{aer}(\mathrm{r})}{\langle\beta_\mathrm{aer}(\mathrm{r})\rangle} + \frac{\delta \sigma^{bac}}{\langle \sigma^{bac}\rangle} \right] m_\mathrm{o}(\mathrm{r,R}) \tag{7}$$

where the dC^bac and σ^bac uncertainties were obtained from the RDG-FA calculations.

Table 1. Mean values of error propagation for the optical and microphysical properties retrieved from lidar. To quantify the error propagation in a single lidar profile, we considered the effect of the radiometric calibration, noise suppression and the inverse method.

**Results - Error propagation**	
**Quantity**	**mean error (%)**
U	6.3
β_{aer}	8.5
n_0	27.6
m_0	24.3

Table 1 summarizes the error propagation results for all quantities retrieved from PSR-EBL technique. For U(r), β,n_0, m_0, the mean errors along the plume are respectively 6.3%, 8.5%, 27.6%, 24.3%. The relative errors on the retrieved concentrations are consistent with other similar lidar studies[8,9].

Supplementary Information 4 - High-resolution time-series from PSR-EBL technique

The PSR-EBL technique performs lidar measurements with spatial resolution of centimeters and temporal resolution of milliseconds. Figure 8 displays the time-series range-averaged mass concentration from the Colibri PSR-EBL instrument that corresponds with the measurements reported in the Figure 3 of the main manuscript. Such time-series are obtained by averaging the PSR-EBL measurements over range. In the inset of the Figure 8, we highlight the region of interest that corresponds to a soot plume measured by the FIDAS 200. The Table of Figure 8 reports time and range-averaged geometrical mean values of number and mass concentration for the time interval of interest (displayed in the inset) retrieved from PSR-EBL along with those measured by the Fidas 200 instrument, for indicative purposes. The standard deviation is referring to the data spreading around the respective mean value. Such deviation should be interpreted as variability of the soot emission and not as the error associated to the product.

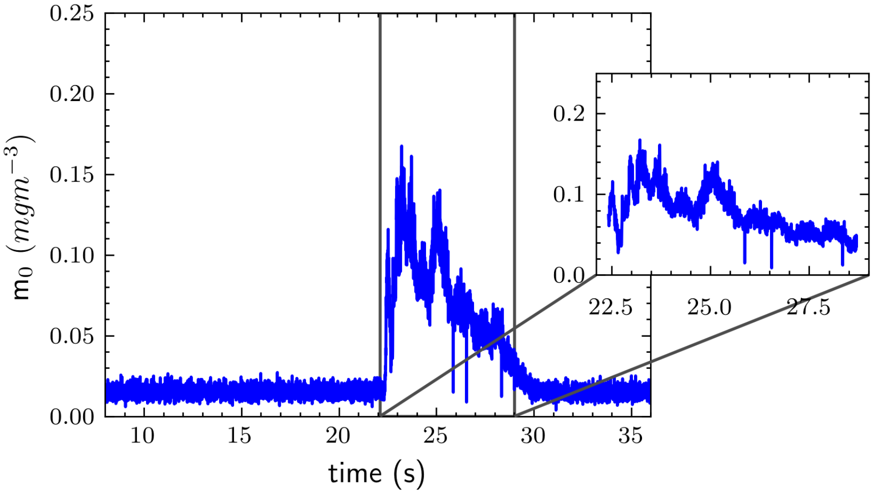

	**FIDAS 200**		**PSR-EBL**	
**Quantity**	**mean**	σ_{geo}	**mean**	σ_{geo}
$\langle n_0 \rangle_{geo}$ [particules/cm^3]	7668.08	1.44	8015.84	2.35
$\langle m_0 \rangle_{geo}$ [mg/m^3]	0.02	2.4	0.07	1.43

Figure 8. Time-series (range-averaged) of mass concentration derived from PSR-EBL measurements for the soot plume displayed in Figure 3 of the main manuscript. The geometric mean values (time and range-averaged) for the number and mass concentration are given for the PSR-EBL technique and for the FIDAS 200 optical particle counter.

Overall, the concentrations measured by the Fidas 200 instrument are within the ranges of the time and range-averaged concentrations retrieved by Colibri instrument. A strict inter-comparison between the two instruments was not possible here because of the unsteady behavior of the turbulent pool fire and because of the differences in time resolution of the two instruments, *i.e.* Fidas 200 operates at four orders of magnitude slower ($\sim$2 min) than Colibri instrument (1 ms). The measurements provided by the optical particle counter are only given here as an indication of the ambient particulate matter number and mass concentration during the soot emission from the pool fire. The salient point is that both optical techniques, in-situ and remote-sensing, provide the same order of magnitude for both number and mass concentration.

References

1. Kelesidis, G. A., Bruun, C. A. & Pratsinis, S. E. The impact of organic carbon on soot light absorption. *Carbon* **172**, 742–749, DOI: https://doi.org/10.1016/j.carbon.2020.10.032 (2021).

2. Paulien, L., Ceolato, R., Soucasse, L., Enguehard, F. & Soufiani, A. Lidar-relevant radiative properties of soot fractal aggregate ensembles. *J. Quant. Spectrosc. & Radiat. Transf.* **241**, 106706 (2020).

3. Liu, L. & Mishchenko, M. I. Spectrally dependent linear depolarization and lidar ratios for nonspherical smoke aerosols. *J. Quant. Spectrosc. Radiat. Transf.* **248**, 106953, DOI: https://doi.org/10.1016/j.jqsrt.2020.106953 (2020).

4. Mazzoleni, C., Kuhns, H. D. & Moosmüller, H. Monitoring automotive particulate matter emissions with lidar: A review. *Remote. Sens.* **2**, 1077–1119, DOI: 10.3390/rs2041077 (2010).

5. Bissonnette, L. R. Lidar and multiple scattering. In *Lidar*, 43–103 (Springer, 2005).

6. Oppel, U. G. & Czerwinski, G. Multiple scattering lidar equations including polarization and change of wavelength. In Atanasov, P. A. & Stoyanov, D. V. (eds.) *Tenth International School on Quantum Electronics: Laser Physics and Applications*, vol. 3571, 14 – 25, DOI: 10.1117/12.347604. International Society for Optics and Photonics (SPIE, 1999).

7. Mishchenko, M. I. *Electromagnetic scattering by particles and particle groups: an introduction* (Cambridge University Press, 2014).

8. Lopatin, A. *et al.* Enhancement of aerosol characterization using synergy of lidar and sun-photometer coincident observations: the garrlic algorithm. *Atmospheric Meas. Tech.* **6**, 2065–2088, DOI: 10.5194/amt-6-2065-2013 (2013).

9. Benavent-Oltra, J. A. *et al.* Different strategies to retrieve aerosol properties at night-time with the grasp algorithm. *Atmospheric Chem. Phys.* **19**, 14149–14171, DOI: 10.5194/acp-19-14149-2019 (2019).
